# Supplementary material for: All-at-once RNA folding with 3D motif prediction framed by evolutionary information
Source: Nat Methods. 2025 Oct 3;22(10):2094–106. doi: 10.1038/s41592-025-02833-w (PMC12510878; doi:10.1038/s41592-025-02833-w)
Supplement: Supplementary file 3 — Source code to run CaCoFold-R3D. Data and results. [file 41592_2025_2833_MOESM3_ESM.gz › supplemental_material/results/CaCoFold-R3D/CaCoFold-R3D_Rfam/RF02166_PVT1_3.R2R.sto.pdf]

RF02166_PVT1_3

5′-Y Y ○ U Y ● ● G R ● R R C Y R Y R C C U G Y C R G Y U R C ● Y G G A G C Y Y Y R Y U Y A R R R ● U U U Y C Y G A G C C U R R U G G A U U U A C A G Y G A U R Y U Y R G Y Y R C C U G R G G A R
